# The Effectiveness of a Functional Preconditioning Program in Reducing Musculoskeletal Injuries in a Tactical Population

**DOI:** 10.3390/sports14070260

**Published:** 2026-06-23

**Authors:** Hamad Alkaabi, Everett Lohman, Mansoor Alameri, Noha Daher, Aleksandar Čvorović, Hatem Jaber

**Affiliations:** 1 Department of Physical Therapy, School of Allied Health Professions, Loma Linda University, Loma Linda, CA 11060, USA; elohman@llu.edu (E.L.); ndaher@llu.edu (N.D.); 2 Department of Physical Therapy, College of Rehabilitative Sciences, University of St. Augustine for Health Sciences, Austin, TX 78739, USA; malameri@usa.edu; 3 Police Sports Education Center, Human Resources Sector, Abu Dhabi Police, Abu Dhabi P.O. Box 253, United Arab Emirates; a.cvorovic@adpolice.gov.ae; 4 Department of sport sciences, College of Higher Vocational Studies “Sports Academy”, 11000 Belgrade, Serbia

**Keywords:** preconditioning, tactical, athlete, functional, basic military training, training, musculoskeletal, injury, prevention, police

## Abstract

This study examined the effects of functional training on musculoskeletal (MSK) injuries, days lost from training, and attrition rate among police recruits in Abu Dhabi. A total of 441 Abu Dhabi Police recruits were randomly allocated into experimental (*n* = 220) and control (*n* = 221) groups. The experimental group underwent six weeks of preconditioning functional training, while the control group followed the standard Abu Dhabi Police recruitment procedure. Subsequently, both groups entered 16 weeks of basic military training (BMT). The outcome measures (injury rate, days lost from training, and attrition rate) were collected at the end of BMT. The proportion of injuries reported by the experimental group was significantly lower than that reported by the control group (15.3% versus 52.3%, respectively [χ^2^ = 66.8, *p* < 0.001]). There were significant differences in the median (minimum, maximum) number of injuries and number of days lost to training between the experimental and control groups [1 (1, 5) versus 2 (1, 9), *p* = 0.002 (r = 0.30); and 2.5 (1, 26) versus 5.0 (1, 37), *p* < 0.001 (r = 0.34), respectively]. The number of dropouts due to MSK injuries during BMT was 1 in the experimental group and 5 in the control group (*p* = 0.06). Tactical athletes who did not undergo preconditioning training demonstrated a greater risk of non-contact lower-limb injury than those who received structured conditioning training. Therefore, preconditioning training might be a beneficial tool to minimize injuries among tactical athlete trainees. Trial Registration: ISRCTN registry (ISRCTN41786994), retrospectively registered on 13 April 2026.

## 1. Introduction

Tactical athletes (TAs) are individuals with specialized physical capabilities who operate in demanding occupational environments, including the military, police, firefighting, and emergency services [1]. TAs require a significant level of functional fitness to perform physically demanding occupational tasks [2]. Consequently, new recruits entering these professions undergo rigorous basic military training (BMT) to prepare for the physical demands of service.

Musculoskeletal (MSK) injuries during BMT are common, with reports indicating that more than 80% of injuries sustained during military training are MSK in nature [3,4]. These injuries account for approximately 60% of all days lost from training [5]. Furthermore, MSK injuries have been identified as the leading cause of attrition during BMT. Attrition rates of approximately 3.5% have been reported, with MSK injuries accounting for nearly 43% of these cases [6]. The high rates of injury and attrition place a substantial burden on police and military institutions. According to a recent report, the United States military spends approximately USD 3000 per musculoskeletal injury, resulting in an estimated annual expenditure of USD 2.5 billion among new recruits [7].

Low physical fitness is one of the most commonly reported risk factors for MSK injuries among tactical athlete trainees [8], particularly poor lower-extremity power, elevated body mass index, and low cardiovascular fitness [9,10]. Therefore, improving physical fitness may be important for reducing the individual, societal, and institutional consequences associated with training-related injuries. Preconditioning training (PCT) programs designed to improve physical fitness have been examined in several studies. For example, Knapik et al. [10] reported that U.S. Army recruits who underwent PCT prior to BMT experienced significantly lower injury and attrition rates. Similarly, a study involving recruits from the Netherlands Armed Forces reported fewer days lost from training among participants who completed a preconditioning program compared with controls [11]. PCT programs have also been investigated in other countries, including Singapore [12]. However, there is limited evidence regarding their effectiveness in tactical athlete populations from Arabian Peninsula countries, such as the United Arab Emirates and Saudi Arabia, where physical inactivity has been reported as a public health concern [13].

Training-related injuries and attrition impose a substantial burden on police and military institutions in Abu Dhabi. Preventing and minimizing MSK injuries is a priority for the Abu Dhabi Police because of their impact on physical readiness and the costs associated with medical treatment, rehabilitation, and days lost from training. As part of an injury prevention initiative, the Abu Dhabi Police Department developed and implemented a PCT program designed to improve recruits’ physical fitness before entering BMT and to evaluate its effectiveness in reducing injury rates, days lost from training, and attrition among Arab tactical athlete trainees. Therefore, the purpose of this study was to evaluate the effectiveness of a PCT program in reducing injury rates, days lost from training, and attrition among Abu Dhabi police recruits.

## 2. Materials and Methods

### 2.1. Study Design

A randomized controlled trial was conducted at the Abu Dhabi Police Training Academy, United Arab Emirates (UAE), between October 2021 and June 2022. The trial was retrospectively registered with the ISRCTN registry (ISRCTN41786994) on 13 April 2026. The study protocol, intervention procedures, outcome measures, and statistical analyses were established prior to participant recruitment and data collection.

### 2.2. Participants

A total of 441 male participants were selected from the Abu Dhabi police recruitment department after passing the recruitment medical screening (RMS). The Abu Dhabi RMS includes a physical exam, blood work, a mental health assessment, a vision exam, and a chest X-ray. Following the RMS, participants attended an orientation session regarding the study and were asked to volunteer if they were interested. All participants read and signed a written informed consent approved by the Institutional Review Board of Abu Dhabi Department of Health (ADDH), UAE [IRB# DOH/CVDC/2021/736] and Loma Linda University Health (LLUH), USA [IRB#5210303] prior to participation. Eligible participants met the following inclusion criteria: (1) age 18–40 years; and (2) passing the RMS. Participants were excluded if they reported musculoskeletal injuries, neurological or vestibular symptoms, or surgeries since passing the RMS.

### 2.3. Procedures and Intervention

In total, 441 participants who met the inclusion criteria were recruited and randomly assigned to either the experimental or control group. Group assignment was conducted by an independent staff member from the Abu Dhabi Police recruitment department using a computerized random number generator and was concealed using sealed envelopes from all personnel involved in participant screening prior to randomization. The allocation ratio was approximately 1:1, resulting in 220 participants in the experimental group and 221 participants in the control group. Four physical therapists with an average of 10 years of tactical athlete clinical experience, who were blinded to group assignment, collected demographic data and performed baseline measurements. The assessors were licensed physical therapists with extensive experience in movement assessment and received training in the administration and scoring of the Functional Movement Screen (FMS), testing sequence, participant instructions, scoring procedures, and measurement protocols prior to study commencement to ensure standardized administration of all assessments. One assessor held FMS certification and supervised FMS testing and scoring throughout the study to ensure adherence to the standardized protocol. Baseline measurements were conducted during scheduled morning testing sessions at the Abu Dhabi Police Training Academy. The Functional Movement Screen (FMS) was administered prior to any warm-up activities in accordance with the standardized FMS protocol to allow assessment of natural movement patterns. Following completion of the FMS, participants performed a standardized warm-up consisting of light aerobic activity and dynamic stretching prior to the remaining physical performance assessments. The assessments were administered in the same sequence for all participants to minimize potential order effects and ensure consistency across testing sessions. Standardized verbal instructions and demonstrations were provided before each assessment, and participants were allowed familiarization trials when required by the testing protocol. Participants were instructed to wear standard athletic clothing and footwear and to provide maximal effort during all physical performance tests. Rest periods of approximately 30–60 s were provided between repeated trials of the same test and approximately 2 min between different assessments to minimize the effects of fatigue.

Demographic data included age, height, weight, body mass index (BMI), and body fat percentage (using a GAIA 359 plus professional, South Korea). The baseline measurements were only taken pre-intervention in order to control for any baseline differences. These measurements included (1) a FMS to assess movement patterns and identify areas of dysfunction or limitations in movement. More specifically, the FMS consists of seven tests that assess mobility, stability, and neuromuscular control, including the deep squat, hurdle step, in-line lunge, active straight-leg raise, trunk stability push-up, rotary stability, and shoulder mobility [14]. The FMS was administered and scored according to the standard protocol described by Cook et al. [15,16], with each movement scored on a 0–3 scale and a composite score ranging from 0 to 21 used for analysis. (2) A handgrip strength test using a Jamar dynamometer to assess grip strength [17,18]. Three maximal trials were performed on each side according to standardized testing procedures, with approximately 30–60 s of rest provided between trials. The mean of the three trials was used for analysis to reduce the influence of trial-to-trial variability. (3) The Y-Balance Test to assess dynamic balance [19,20]. Three successful trials were performed in each reach direction, and the composite score was used for analysis. (4) The self-report International Physical Activity Questionnaire (IPAQ) to assess physical activity level [21]. (5) Measurement of lower extremity strength using a Back–Leg–Chest Dynamometer (BLCD) (TKK 5402, Takei Scientific Instruments Co., Ltd., Niigata, Japan) [22]. Three maximal efforts were performed with approximately 30–60 s of rest between trials. The mean of the three trials was used for analysis to reduce the influence of trial-to-trial variability. (6) A vertical jump test using the Vertec Jump device (Vertec, Sports Imports, Hilliard, OH, USA) to assess lower body power and explosiveness [23]. Three maximal jump attempts were performed with approximately 30–60 s of recovery between trials. The mean jump height was recorded and used for analysis to reduce the influence of trial-to-trial variability.

Following the collection of the baseline data, the experimental group entered a 6-week PCT program, while the control group was instructed to wait for a 6-week period prior to entering the BMT following the current standard Abu Dhabi Police recruitment procedure (no further instructions were given). Experienced fitness trainers (in consultation with the study authors) designed and administered the PCT program for the experimental group. Prior to implementation, all trainers received orientation regarding the study protocol, exercise progression, training objectives, load progression guidelines, and session procedures to ensure standardized delivery of the intervention. Program implementation was monitored throughout the intervention period by experienced exercise and fitness personnel to ensure adherence to the prescribed protocol (A detailed description of the preconditioning training protocol is provided in the Supplementary Material).

**Figure 1:**
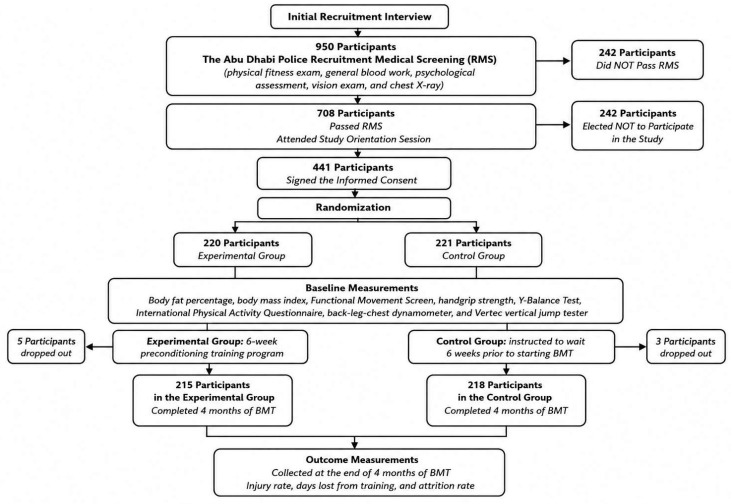
Study procedure flowchart.

The PCT consisted of 6 weeks of high-intensity functional training exercises. The first two weeks consisted of exercises aimed at improving muscle activation and flexibility; the third and fourth weeks included exercises to improve endurance and strength; and the last two weeks included exercises to enhance strength and power (Table 1). Load management, including how much each subject trained, performed, and rested, was accounted for to gradually achieve the optimal load. The study utilized bodyweight exercises with load adjustments based on the Rating of Perceived Exertion (RPE) scale [24], progressively increasing the load level week by week. The weekly training load consisted of four training units, including two days of training, one day of rest, two more days of training, then two days of rest. In each two-day block, the first training day focused on strength training, while the second focused on cardiorespiratory system training. The Tabata method was often used to adjust the prescribed loads to individual capabilities while maintaining the intended progression framework. Overall, the training program was designed to prepare recruits for upcoming efforts during basic military training and focus on movement quality to minimize possible injuries. Attendance and participation in the PCT program were monitored by the Abu Dhabi Police Physical Fitness Administration in accordance with standard training procedures. Prior to participation, recruits were informed of the program requirements and attendance expectations. Participants who missed more than three training sessions were excluded from the program. No participant exceeded this threshold, and all recruits assigned to the experimental group successfully completed the 6-week PCT program. Any absences or withdrawals during the intervention period were documented and reported to the research team for follow-up. Participants who withdrew prior to entering Basic Military Training were accounted for in the study flowchart (Figure 1).

### 2.4. Outcome Measures

The outcome variables were obtained from the Abu Dhabi Police Academy medical clinic following completion of BMT. The primary outcomes included injury rate, days lost from training, and attrition due to MSK injuries (Figure 1). All injury diagnoses, medical evaluations, training restrictions, exemptions, days lost from training, and attrition decisions were determined and documented by medical personnel at the Police Academy medical clinic as part of the routine medical management process.

For the purposes of this study, an injury was defined as a musculoskeletal condition documented by the medical clinic that resulted in medical evaluation and/or modification of training participation. Days lost from training were defined as the number of training days missed due to medically prescribed restrictions associated with an MSK injury. The research team subsequently received and analyzed the medical records. Acute and overuse injuries were not analyzed separately because the primary objective of the study was to evaluate the overall impact of the preconditioning training program on MSK injury occurrence, days lost from training, and attrition during BMT.

### 2.5. Data Analyses

Data were analyzed using SPSS version 28.0 (IBM, Chicago, IL, USA). A priori sample size was estimated using G*Power (Version 3.1, Heinrich Heine University Düsseldorf, Düsseldorf, Germany). In the absence of directly comparable studies reporting effect sizes for the intervention and outcomes investigated, a moderate effect size (Cohen’s d = 0.50) was selected based on Cohen’s recommendations for sample size estimation [25]. Considering a moderate effect size of 0.5, a power of 0.80, an alpha level of 0.05, and a 20% dropout rate, the estimated sample size was 400 participants. The data are summarized using frequency (%) for qualitative variables, mean and standard deviation (SD) for continuous variables, or median (minimum, maximum) when the distribution was not approximately normal. Normality of the outcome variables was examined using the Shapiro–Wilk test. Mean (SD) of participants’ characteristics and baseline measures by study group were compared using an independent *t*-test. The number of injuries, pre-military training dropouts, and military training dropouts due to MSK injury by study group was assessed using Fisher’s Exact test. The median (minimum, maximum) number of injuries and days lost from training were compared between the two groups using the Mann–Whitney U test. Among participants who reported at least one injury, the distribution of leave type and injury location by study group was examined using Fisher’s Exact test.

Effect sizes were reported according to the statistical test performed. Cohen’s d was used for comparisons, analyzed using independent *t*-tests and effect size r was calculated for Mann–Whitney U tests. Cohen’s d values were interpreted as small (0.20), medium (0.50), and large (0.80), while r values were interpreted as small (0.10), medium (0.30), and large (0.50), according to Cohen’s recommendations [25]. For categorical outcomes analyzed using Fisher’s Exact tests, odds ratios (ORs) and corresponding 95% confidence intervals (CIs) were reported to quantify the magnitude and direction of associations between groups.

The primary study outcomes were injury incidence, days lost from training, and attrition due to musculoskeletal injury. Analyses of leave categories and injury locations were considered secondary exploratory analyses intended to provide additional clinical information regarding injury characteristics. Therefore, adjustments for multiple comparisons were not applied. Findings from secondary analyses should be interpreted cautiously because multiple statistical comparisons may increase the risk of Type I error. The level of significance was set at *p* < 0.05.

## 3. Results

A total of 441 males provided informed consent and were randomly assigned to the experimental (*n*_1_ = 220) or control group (*n*_2_ = 221). Five participants from the experimental group and three from the control group withdrew prior to BMT due to non-MSK-related injuries (Figure 1). Consequently, 433 males aged 21.7 ± 2.5 years (mean ± SD), with a BMI of 24.4 ± 5.7 kg/m^2^ and body fat percentage of 21.9 ± 8.0%, participated in the study. There were no significant differences between the study groups with respect to age, height, weight, BMI, body fat percentage, or baseline outcome measures (*p* > 0.05) (Table 2 and Table 3).

The proportion of recruits reporting at least one injury during BMT was significantly lower in the experimental group than in the control group. Only 15.3% (*n* = 33) of recruits in the experimental group reported at least one injury compared with 52.3% (*n* = 114) in the control group [χ^2^ = 66.8, *p* < 0.001; OR = 0.16; 95% CI (0.10, 0.26); Table 4]. This finding indicates that recruits in the experimental group had approximately 84% lower odds of reporting at least one injury compared with controls. Attrition due to MSK injuries during BMT was also lower in the experimental group, with 1 subject withdrawing compared with 5 participants in the control group; however, this difference did not reach statistical significance (*p* = 0.06; Table 4).

A total of 147 participants (33.9%) reported at least one injury. Among these individuals, the frequency of injuries and the number of days lost from training were compared between groups. Significant differences were observed for both outcomes. The median (min, max) number of injuries was lower in the experimental group than in the control group [1 (1, 5) versus 2 (1, 9), *p* = 0.002, r = 0.30]. Similarly, the median (min, max) number of days lost from training was lower in the experimental group [2.5 (1, 26) versus 5.0 (1, 37), *p* < 0.001, r = 0.34] (Table 5).

When leave categories (day of rest, exempt from sport, exempt from military, and exempt from running) were compared between groups, the proportions were consistently lower in the experimental group. The proportion of recruits requiring a day of rest was significantly lower in the experimental group than in the control group [42.4% versus 67.5%, χ^2^ = 6.8, *p* = 0.008; OR = 0.35; 95% CI (0.16, 0.78)]. Although the proportions of recruits receiving exemptions from sport, military activities, and running were also lower in the experimental group, these differences were not statistically significant (*p* > 0.05) (Table 6).

When injury location was examined, lower proportions were observed in the experimental group across most injury categories. The proportion of recruits reporting hip and spine injuries was significantly lower in the experimental group than in the control group [6.1% versus 23.7%, χ^2^ = 5.0, *p* = 0.017; OR = 0.21; 95% CI (0.05, 0.93)]. Although the proportions of knee and thigh injuries and upper-limb injuries were also lower in the experimental group, these differences were not statistically significant (*p* > 0.05) (Table 6).

## 4. Discussion

The burden of musculoskeletal injuries during basic military training remains a significant challenge for police and military organizations worldwide. Although pre-conditioning programs have been investigated in several military populations, there is limited evidence regarding their effectiveness among police recruits from the Arabian Peninsula, particularly within the Abu Dhabi Police training system. Therefore, this study was conducted to address this gap and evaluate the potential benefits of implementing a structured preconditioning program prior to basic military training. This study examined the effects of 6 weeks of PCT on injury rates, days lost from training, and attrition among Abu Dhabi police trainees. The results suggest that participation in PCT may be associated with lower rates of MSK injuries and their related consequences among police trainees. In particular, recruits who underwent PCT prior to BMT experienced fewer MSK injuries and fewer days lost from training than those who did not. Although the difference in attrition rate was not statistically significant, recruits who underwent PCT demonstrated a lower tendency to drop out.

Several studies have reported that PCT programs administered prior to BMT are associated with lower injury and attrition rates. Knapik et al. examined the effects of PCT on injury rates and attrition among different groups of U.S. military recruits [10] and reported lower injury and attrition rates among recruits who underwent preconditioning compared with those who did not. Similarly, Lee et al. assessed the effects of 4–6 weeks of PCT on attrition among Singaporean military trainees and found lower attrition rates among trainees who underwent preconditioning. In that study, MSK injuries sustained during BMT were identified as a major contributor to attrition [12]. In contrast, a recent study from the Netherlands by Dijksma et al. reported higher rates of injury and dropout due to MSK injuries in the preconditioning group [11]. However, the authors noted several methodological limitations, including a relatively small sample size and missing data, which may have influenced their findings.

The findings of this study are generally consistent with previously published research. However, the present study extends the existing literature by providing evidence from a tactical population that has been underrepresented in previous research. Unlike most prior investigations conducted in military settings, this study evaluated a structured preconditioning program in a police training environment, providing practical information that may assist police and tactical organizations in the region when developing injury prevention strategies. Given the limited availability of data from police and tactical populations in the Arabian Peninsula, these findings contribute region-specific evidence that may support the development of training and injury prevention initiatives in similar settings. The substantial reduction in injury rates and days lost from training observed in the present study suggests that structured preconditioning programs may represent a practical approach to improving recruit readiness and reducing the burden of musculoskeletal injuries during basic military training.

Training-related injuries may negatively impact police trainees and impose a considerable burden on police institutions. Injuries can result in physical limitations and disability, which may compromise military readiness. Low physical fitness and low muscular endurance among recruits have been associated with an increased risk of injury and attrition [10,26]. Furthermore, mental health-related problems such as stress and depressive symptoms have been identified as contributors to attrition during BMT [27,28]. BMT is typically physically and psychologically demanding for new recruits who are transitioning into a rigorous training environment. The physical and mental demands of BMT may place considerable stress on trainees and increase their susceptibility to adverse psychological strain [29]. Previous research has also shown that recruits with lower levels of physical fitness may be more susceptible to depressive symptoms during training than those with moderate to high levels of fitness [30,31]. Therefore, building physical endurance and strength prior to entering BMT may better prepare recruits for the physical demands associated with military training. The lower injury rates and fewer days lost from training observed among recruits who underwent PCT suggest that the program may have enhanced readiness for BMT. However, because physiological and psychological adaptations were not directly assessed, the mechanisms underlying these findings cannot be definitively determined.

The PCT program was designed to improve physical fitness and endurance prior to entering BMT. It focused primarily on the progressive development of strength, endurance, mobility, and movement quality while providing sufficient recovery between training sessions. Previous research has demonstrated considerable variability in individual responses to similar exercise programs, particularly with respect to improvements in endurance performance, strength, and mobility [32]. Therefore, the program was structured to gradually increase training demands over the 6-week period, allowing recruits to progressively adapt to the physical requirements associated with BMT. The inclusion of progressive loading, recovery periods, and movement-focused exercises was intended to facilitate a safe transition into the more demanding training environment encountered during BMT. Overall, the program was designed to provide military and police institutions with a standardized, safe, and structured approach to preparing recruits for the physical demands of training and potentially reducing the risk of training-related injuries.

Study Limitations: The physical activity level of the control group was not tracked during the 6-week waiting period prior to BMT. Thus, it is unknown whether any participants in the control group participated in additional training during this period. However, the control group reflected the standard Abu Dhabi Police recruitment procedure, which was the intended comparator in the present study. Despite any potential variation in physical activity during this period, recruits who participated in the structured PCT program experienced substantially lower injury rates and fewer days lost from training during BMT than recruits who followed the standard recruitment pathway. Likewise, adherence to the prescribed PCT program and any additional physical activity performed outside of the intervention were not formally monitored. These factors may have influenced the observed outcomes and should be considered when interpreting the results. Additionally, women were not included in this study due to logistical limitations (e.g., feasibility). Therefore, future studies should investigate potential sex-related differences in response to PCT. Another limitation is that physical performance and baseline fitness measures were not reassessed following completion of the 6-week PCT program. Consequently, changes resulting from the intervention could not be quantified, limiting our ability to determine the physiological adaptations that may have contributed to the observed reductions in injury rates and days lost from training. The nutritional or dietary status of participants was also not monitored during the intervention period and may have influenced training outcomes. Furthermore, psychosocial factors that may contribute to injury risk or attrition were not assessed. Additional limitations include the retrospective registration of the trial and the lack of follow-up after completion of BMT, which prevented examination of the long-term effects of PCT and whether the observed benefits were maintained over time.

## 5. Conclusions

The results of the present study suggest that participation in a PCT program prior to BMT may be associated with lower rates of training-related injuries and fewer days lost from training, with a trend toward reduced attrition due to MSK injuries. The greater reduction in injury rates observed in the present study compared with some previous reports may be related to differences in participant characteristics, baseline fitness levels, training protocols, and the specific demands of Abu Dhabi Police basic military training. Additionally, recruits may have benefited from a structured, progressive preconditioning program tailored to their physical readiness prior to entering BMT. However, given the limitations of the study, including the lack of post-intervention physical performance testing and adherence monitoring, the mechanisms underlying these findings cannot be definitively determined.

This study provides evidence supporting the potential value of structured preconditioning programs within a police training setting and adds to the limited literature available from tactical populations in the Arabian Peninsula. The findings suggest that implementation of a structured preconditioning program may reduce musculoskeletal injuries and training disruption among Abu Dhabi Police recruits. As such, these results may help inform injury prevention initiatives and training practices aimed at improving recruit readiness while reducing the operational and financial burden associated with injuries sustained during basic military training in similar police and military settings.

Overall, PCT appears to be a promising strategy for preparing recruits for the physical demands of BMT and may contribute to reduced injury risk and training disruption. Future prospective studies are needed to confirm these findings, examine the mechanisms responsible for the observed outcomes, and determine the long-term effects of PCT. Future studies should also assess psychosocial factors and their interaction with physical factors, as well as evaluate potential modifications to existing BMT programs that may further reduce injury risk and attrition.

## 6. Clinical Application

The results of this study have practical relevance for BMT personnel. Recruits who underwent PCT demonstrated lower rates of MSK injuries, fewer days lost from training, and lower attrition compared with recruits who did not undergo preconditioning. Therefore, implementation of a structured PCT program may be considered as a potential strategy to prepare recruits for the physical demands of BMT and reduce training-related injuries. However, additional prospective research is needed to confirm these findings and establish best-practice recommendations.

## Figures and Tables

**Table 1:** Preconditioning training protocol.

Six-Week General Training Plan									
Phase	Week	Sun.	Mon.	Tue.	Wed.	Thu.	Fri.	Sat.	Main Goals
1. Activation phase	1	Strength-isometric (Tabata circuit)	Cardio walk–run (interval)	Off	Strength-isometric (Tabata circuit)	Cardio walk–run (interval)	Off	Off	Isometric strength development and core stability Running technique Optimal mobility and flexibility
	2	Strength-isometric (Tabata circuit)	Cardio pace–run (interval)	Off	Strength-isometric (Tabata circuit)	Cardio steady-state run	Off	Off	
2. Endurance and strength phase	3	Strength-endurance (Tabata circuit)	Cardio-agility Tabata	Off	Strength-endurance (work at stations)	Cardio pace–run (interval)	Off	Off	Local muscular endurance Aerobic capacity Agility development (change of direction speed)
	4	Strength-endurance (Tabata circuit)	Cardio-agility Tabata	Off	Strength-endurance (work at stations)	Cardio steady-state run	Off	Off	
3. Strength and power phase	5	Strength-hypertrophy. Work at stations	Cardio-agility Tabata	Off	Strength and low-impact plyometrics	Cardio pace–run (interval)	Off	Off	Hypertrophy Aerobic and anaerobic capacity Low to medium plyometrics
	6	Strength-hypertrophy. Work at stations	Cardio-agility Tabata	Off	Strength and low-impact plyometrics	Cardio steady-state run	Off	Off	

**Table 2:** Mean (SD) of characteristics of participants by study group.

	Experimental (*n*_1_ = 220)	Control (*n*_2_ = 221)	*p*-Value (Cohen’s *d*)
Age (years)	21.6 (2.5)	21.8 (2.5)	0.39 (0.08)
Height (cm)	172.6 (5.7)	172.1 (5.6)	0.40 (0.09)
Body Mass (kg)	73.0 (16.0)	71.7 (14.1)	0.41 (0.09)
BMI (kg/m^2^)	24.6 (6.8)	24.2 (4.4)	0.41 (0.08)
Body Fat %	22.5 (9.1)	21.4 (6.6)	0.17 (0.14)
Pre-Military Training Dropout, *n* (%)	5 (2.3)	3 (1.4)	0.47 (1.05)

Abbreviation: SD, standard deviation; BMI, body mass index.

**Table 3:** Mean (SD) of baseline measurements.

	Experimental (*n*_1_ = 220)	Control (*n*_2_ = 221)	*p*-Value (Cohen’s *d*)
Functional Movement Screen (score)	14.3 (1.4)	14.2 (1.3)	0.20 (0.07)
Hand Grip (kg)	39.8 (6.68)	40.8 (8.1)	0.16 (0.13)
Right Y Balance (cm)	104.8 (5.8)	105.4 (5.8)	0.26 (0.10)
Left Y Balance (cm)	105.5(8.0)	105.6 (5.7)	0.87 (0.01)
Back–Leg–Chest (kg)	129.2 (14.9)	126.7 (28.6)	0.40 (0.08)
Vertical Jump (cm)	46.7 (6.9)	45.5 (7.4)	0.14 (0.18)

Abbreviation: SD, standard deviation.

**Table 4:** Frequency (%) of injury and dropout of participants by study group (*n* = 433).

	Experimental (*n*_1_ = 215)	Control (*n*_2_ = 218)	*p*-Value *	Odds Ratio (95% CI)
Reported Injury	33 (15.3)	114 (52.3)	<0.001	0.16 (0.10, 0.26)
Military Training Dropout (MSK Injury)	1 (0.5)	5 (2.3)	0.11	0.20 (0.02, 1.72)

Abbreviation: MSK, musculoskeletal; * Fisher’s Exact test.

**Table 5:** Median (minimum, maximum) for frequency of injury and days lost among injured participants by study group (*n* = 147).

	Experimental (*n*_1_ = 33)	Control (*n*_2_ = 114)	*p*-Value * (r)
Number of injuries	1 (1, 5)	2 (1, 9)	0.002 (0.30)
Number of days lost	3 (1, 26)	5 (1, 37)	<0.001 (0.34)

* Mann–Whitney U test.

**Table 6:** Comparison of frequency (%) of outcome variables among injured participants by study group (*n* = 147).

		Group Type				
		Experimental (*n*_1_ = 33)	Control (*n*_2_ = 114)	Total	*p*-Value *	Odds Ratio (95% CI)
Leave Type	Had a day of Rest (yes)	14 (42.4)	77 (67.5)	91	0.008	0.35 (0.16, 0.78)
	Exempt from Sport (yes)	22 (66.7)	93 (85.6)	115	0.059	0.34 (0.13, 0.91)
	Exempt from Military (yes)	22 (66.7)	88 (77.2)	110	0.159	0.59 (0.25, 1.42)
	Exempt from Running (yes)	22 (66.7)	84 (73.7)	106	0.280	0.72 (0.30, 1.72)
Injury Location	Foot	18 (54.5)	62 (54.4)	80	0.573	1.00 (0.46, 2.18)
	Shin and Calf	8 (24.2)	18 (15.8)	26	0.192	1.70 (0.67, 4.33)
	Knee and Thigh	13 (39.4)	53 (46.5)	66	0.302	0.75 (0.34, 1.65)
	Hip and Spine	2 (6.1)	27 (23.7)	29	0.017	0.21 (0.05, 0.93)
	Upper Limb	2 (6.1)	12 (10.5)	14	0.350	0.55 (0.12, 2.58)

* Fisher’s Exact test.

## Data Availability

The data presented in this study are available on request from the corresponding author due to privacy and ethical restrictions associated with participant information and institutional regulations.

## Supplementary Materials

The following supporting information can be downloaded at: https://www.mdpi.com/article/10.3390/sports14070260/s1.
