# Supplementary material for: The Effectiveness of a Functional Preconditioning Program in Reducing Musculoskeletal Injuries in a Tactical Population
Source: Sports (Basel). 2026 Jun 23;14(7):260. doi: 10.3390/sports14070260 (PMC13418989; doi:10.3390/sports14070260)
Supplement: Supplementary file 1 [file sports-14-00260-s001.zip › sports-4307746-supplementary.pdf]

GENERAL TRAINING PLAN: 6-WEEK, 24 TRAINING UNITS (DRAFT)									
**PHASE**	**WEEK**	**SUN.**	**MON.**	**TUE.**	**WED.**	**THU.**	**FRI.**	**SAT.**	**MAIN GOALS**
**1. ACTIVATION**	**1**	STRENGTH-ISOMETRIC (TABATA CIRCUIT)	CARDIO WALK-RUN (INTERVAL)	OFF	STRENGTH-ISOMETRIC (TABATA CIRCUIT)	CARDIO WALK-RUN (INTERVAL)	OFF	OFF	1. ISOMETRIC STRENGTH DEVELOPMENT AND CORE STABILITY; 2. RUNNING TECHNIQUE; 3. OPTIMAL MOBILITY AND FLEXIBILITY
	**2**	STRENGTH-ISOMETRIC (TABATA CIRCUIT)	CARDIO PACE-RUN (INTERVAL)	OFF	STRENGTH-ISOMETRIC (TABATA CIRCUIT)	CARDIO STEADY-STATE RUN	OFF	OFF	
**2. ENDURANCE AND STRENGTH PHASE**	**3**	STRENGTH-ENDURANCE (TABATA CIRCUIT)	CARDIO-AGILITY TABATA	OFF	STRENGTH-ENDURANCE (WORK AT STATIONS)	CARDIO PACE-RUN (INTERVAL)	OFF	OFF	1. LOCAL MUSCULAR ENDURANCE; 2. AEROBIC CAPACITY; 3. AGILITY DEVELOPMENT (CHANGE OF DIRECTION SPEED)
	**4**	STRENGTH-ENDURANCE (TABATA CIRCUIT)	CARDIO-AGILITY TABATA	OFF	STRENGTH-ENDURANCE (WORK AT STATIONS)	CARDIO STEADY-STATE RUN	OFF	OFF	
**3. STRENGTH AND POWER PHASE**	**5**	STRENGTH-HYPERTROPHY WORK AT STATIONS	CARDIO-AGILITY TABATA	OFF	STRENGTH AND LOW IMPACT PLYO.	CARDIO PACE-RUN (INTERVAL)	OFF	OFF	1. HYPERTHROPHY; 2. AEROBIC AND ANAEROBIC CAPACITY; 3. LOW TO MEDIUM PLYOMETRICS;
	**6**	STRENGTH-HYPERTROPHY WORK AT STATIONS	CARDIO-AGILITY TABATA	OFF	STRENGTH AND LOW IMPACT PLYO.	CARDIO STEADY-STATE RUN	OFF	OFF	

Comments: The primary goal of this program is to prepare cadets for the challenges they will face during their training at the Police College. Because the course is both mentally and physically demanding, this approach is intended to reduce the risk of injury during training. The program is functional in nature and focuses on body-weight exercises performed in outdoor conditions. The main part of the training focuses on strength and aerobic endurance, while functional mobility is addressed during the introductory part and flexibility during the final part of each session. Each training session starts with a warm-up and ends with a cool-down.

		SUNDAY					MONDAY			
WEEK		Isometric strength					CARDIO			
		Main part	Tabata Circuit		RPE		Main part	Tabata Circuit		RPE
	#	Exercises	work	REST	Moderate	#	Exercises	work	REST	Moderate
	1	wall sit	20 sec	10 sec	Moderate	1	Spot-up run	20 sec	10 sec	Moderate
	2	front plank	20 sec	10 sec	Moderate	2	Butt kicks	20 sec	10 sec	Moderate
	3	glute bridge	20 sec	10 sec	Moderate	3	Run 5 + 5 m U turn	20 sec	10 sec	Moderate
1	4	side lunge static left	20 sec	10 sec	Moderate	4	Backpedal	20 sec	10 sec	Moderate
	5	side plank left	20 sec	10 sec	Moderate	5	Side shuffle (5 m left–right)	20 sec	10 sec	Moderate
	6	side lunge static right	20 sec	10 sec	Moderate	6	Mountain climber (wall)	20 sec	10 sec	Moderate
	7	side lunge static left	20 sec	10 sec	Moderate	7	Jumping jacks	20 sec	10 sec	Moderate
	8	isometric hang 45	20 sec	10 sec	Moderate	8	Run-backpedal (5 m)	20 sec	10 sec	Moderate
	4 rounds with 2 minutes rest between rounds					4 rounds with 2 minutes rest between rounds				
WEEK		Isometric strength					CARDIO			
		Main part	Tabata Circuit		RPE		Main part	Tabata Circuit		RPE
	#	Exercises	work	REST	Moderate	#	Exercises	work	REST	Moderate
	1	wall sit	20 sec	10 sec	Moderate	1	Spot-up run	20 sec	10 sec	Moderate
	2	front plank	20 sec	10 sec	Moderate	2	Butt kicks	20 sec	10 sec	Moderate
	3	glute bridge	20 sec	10 sec	Moderate	3	Run 5 + 5 m U turn	20 sec	10 sec	Moderate
2	4	side lunge static left	20 sec	10 sec	Moderate	4	Backpedal	20 sec	10 sec	Moderate
	5	side plank left	20 sec	10 sec	Moderate	5	Side shuffle (5 m left–right)	20 sec	10 sec	Moderate
	6	side lunge static right	20 sec	10 sec	Moderate	6	Mountain climber (wall)	20 sec	10 sec	Moderate
	7	side lunge static left	20 sec	10 sec	Moderate	7	Jumping jacks	20 sec	10 sec	Moderate
	8	isometric hang 45	20 sec	10 sec	Moderate	8	Run-backpedal (5 m)	20 sec	10 sec	Moderate
	5 rounds with 2 minutes rest between rounds					5 rounds with 2 minutes rest between rounds				

		WEDNESDAY					THURSDAY			
WEEK		Isometric strength					CARDIO			
		Main part	Tabata Circuit		RPE		Main part	Tabata Circuit		RPE
	#	Exercises	work	REST	Moderate	#	Exercises	work	REST	Moderate
	1	wall sit	20 sec	10 sec	Moderate	1	Spot-up run	20 sec	10 sec	Moderate
	2	front plank	20 sec	10 sec	Moderate	2	Butt kicks	20 sec	10 sec	Moderate
	3	glute bridge	20 sec	10 sec	Moderate	3	Run 5 + 5 m U turn	20 sec	10 sec	Moderate
1	4	side lunge static left	20 sec	10 sec	Moderate	4	Backpedal	20 sec	10 sec	Moderate
	5	side plank left	20 sec	10 sec	Moderate	5	Side shuffle (5 m left–right)	20 sec	10 sec	Moderate
	6	side lunge static right	20 sec	10 sec	Moderate	6	mountain climber (wall)	20 sec	10 sec	Moderate
	7	side lunge static left	20 sec	10 sec	Moderate	7	jumping jacks	20 sec	10 sec	Moderate
	8	isometric hang 45	20 sec	10 sec	Moderate	8	run-backpedal (5 m)	20 sec	10 sec	Moderate
		4 rounds with 90 seconds rest between rounds					4 rounds with 2 minutes rest between rounds			
WEEK		Isometric strength					CARDIO-STEADY STATE			
		Main part	Tabata Circuit		RPE	#	Exercises	TIME		RPE
	#	Exercises	work	REST	Moderate					
	1	wall sit	20 sec	10 sec	Moderate					
	2	front plank	20 sec	10 sec	Moderate					
	3	glute bridge	20 sec	10 sec	Moderate					
2	4	side lunge static left	20 sec	10 sec	Moderate	1	STEADY-STATE RUN	20 Minutes		Moderate
	5	side plank left	20 sec	10 sec	Moderate					
	6	side lunge static right	20 sec	10 sec	Moderate					
	7	side lunge static left	20 sec	10 sec	Moderate					
	8	isometric hang 45	20 sec	10 sec	Moderate					
		5 rounds with 90 seconds rest between rounds								

		SUNDAY						MONDAY			
WEEK		Dynamic strength						CARDIO-AGILITY			
		Main part	Tabata Circuit-STRENGTH					Main part	CARDIO-AGILITY TABATA		
	#	Exercises	work	REST	RPE	Tempo	#	Exercises	work	REST	RPE
	1	Air squat	20 sec	10 sec	Hard	2_2	1	A-skip	20 sec	10 sec	Hard
	2	Push-up (knee)	20 sec	10 sec	Hard	2_2	2	Lateral shuffle (5 m–5 m)	20 sec	10 sec	Hard
	3	Curl-up-Back Bridge	20 sec	10 sec	Hard	2_2	3	Backpedal (10 m)	20 sec	10 sec	Hard
3	4	Vertical pull (assisted)	20 sec	10 sec	Hard	2_2	4	Carioca (5 m–5 m)	20 sec	10 sec	Hard
	5	Back lunge (alternate)	20 sec	10 sec	Hard	2_2	5	8-turn (10 m left–right)	20 sec	10 sec	Hard
	6	Wall push (Shoulders)	20 sec	10 sec	Hard	2_2	6	Zig-zag drill F (5 cones, 2 m)	20 sec	10 sec	Hard
	7	Russian Twist (Feet on floor)	20 sec	10 sec	Hard	2_2	7	Zig-zag drill B (5 cones, 2 m)	20 sec	10 sec	Hard
	8	Horizontal pull (45 degrees)	20 sec	10 sec	Hard	2_2	8	T-drill (10 m–5 m–5 m)	20 sec	10 sec	Hard
		4 rounds with 2 minutes rest between rounds						4 rounds with 2 minutes rest between rounds			
WEEK		Dynamic strength						CARDIO-AGILITY			
		Main part	Tabata Circuit-STRENGTH					Main part	CARDIO-AGILITY TABATA		
	#	Exercises	work	REST	RPE	Tempo	#	Exercises	work	REST	RPE
	1	Air squat	20 sec	10 sec	Hard	2_2	1	A-skip	20 sec	10 sec	Hard
	2	Push-up (knee)	20 sec	10 sec	Hard	2_2	2	Lateral shuffle (5 m–5 m)	20 sec	10 sec	Hard
	3	Curl-up-Back Bridge	20 sec	10 sec	Hard	2_2	3	Backpedal (10 m)	20 sec	10 sec	Hard
4	4	Vertical pull (assisted)	20 sec	10 sec	Hard	2_2	4	Carioca (5 m–5 m)	20 sec	10 sec	Hard
	5	Back lunge (alternate)	20 sec	10 sec	Hard	2_2	5	8-turn (10 m left–right)	20 sec	10 sec	Hard
	6	Wall push (Shoulders)	20 sec	10 sec	Hard	2_2	6	Zig-zag drill F (5 cones, 2 m)	20 sec	10 sec	Hard
	7	Russian Twist (Feet on floor)	20 sec	10 sec	Hard	2_2	7	Zig-zag drill B (5 cones, 2 m)	20 sec	10 sec	Hard
	8	Horizontal pull (45 degrees)	20 sec	10 sec	Hard	2_2	8	T-drill (10 m–5 m–5 m)	20 sec	10 sec	Hard
		5 rounds with 2 minutes rest between rounds						4 rounds with 90 seconds rest between rounds			

	WEDNESDAY						THURSDAY					
WEEK	Strength Endurance						CARDIO-PACE RUN (INTERVAL)					
3		Main part	WORK AT STATIONS					Main part	CARDIO-AGILITY TABATA			
	#	Exercises	REPS	REST	RPE	Tempo	#	Exercises or Activity	PACE	REST	RPE	
	1	Air squats	12 to 15	60 sec	Hard	2_2	1	Interval run 7 x 400 METERS	1:50-2:00 (min:sec) per Interval	2 minutes between intervals	Hard	
	2	Push-up (from knee optional)	12 to 15	60 sec	Hard	2_2						
	3	Sit-ups	12 to 15	60 sec	Hard	2_2						
	4	Horizontal pull (45 degrees)	12 to 15	60 sec	Hard	2_2						
	5	Front lunge (alternate)	12 to 16	60 sec	Hard	2_2						
	6	Shoulder press (Wall)	12 to 15	60 sec	Hard	2_2						
	7	Standing Pull (vertical bar)	12 to 15	60 sec	Hard	2_2						
	8	Low-back extensions	12 to 15	60 sec	Hard	2_2						
	2 SETS PER STATION											
WEEK	Strength Endurance							CARDIO-STEADY STATE				
4		Main part	WORK AT STATIONS				#	Exercises	TIME		Tempo	
	#	Exercises	REPS	REST	RPE	Tempo						
	1	Air squats	12 to 15	60 sec	Hard	2_2	1	STEADY-STATE RUNAT 60-70% OF MAXIMAL HEART RATE IN OUTDOOR CONDITIONS	25 Minutes		Hard	
	2	Push-up (from knee optional)	12 to 15	60 sec	Hard	2_2						
	3	Sit-ups	12 to 15	60 sec	Hard	2_2						
	4	Horizontal pull (45 degrees)	12 to 15	60 sec	Hard	2_2						
	5	Front lunge (alternate)	12 to 16	60 sec	Hard	2_2						
	6	Shoulder press (Wall)	12 to 15	60 sec	Hard	2_2						
	7	Standing Pull (vertical bar)	12 to 15	60 sec	Hard	2_2						
	8	Low-back extensions	12 to 15	60 sec	Hard	2_2						
	3 SETS PER STATION											

	SUNDAY						MONDAY				
WEEK	STRENGTH (HYPERTHROPHY) WORK AT STATIONS						CARDIO-AGILITY				
5		Main part	Tabata Circuit-STRENGTH					Main part	CARDIO-AGILITY TABATA		
	#	Exercises	REPS	REST	RPE	Tempo	#	Exercises	work	REST	RPE
	1	Prisoner squat	8 to 12	60 sec	Very hard	2_1	1	A-skip	20 sec	10 sec	Very hard
	2	Push-up	8 to 12	60 sec	Very hard	2_1	2	Lateral shuffle (5 m–5 m)	20 sec	10 sec	Very hard
	3	Sit-ups	8 to 12	60 sec	Very hard	2_1	3	Backpedal (10 m)	20 sec	10 sec	Very hard
	4	Vertical pull (assisted)	8 to 12	60 sec	Very hard	2_1	4	Carioca (5 m–5 m)	20 sec	10 sec	Very hard
	5	Front lunge (alternate)	8 to 12	60 sec	Very hard	2_1	5	8-turn (10 m left–right)	20 sec	10 sec	Very hard
	6	Shoulder press (floor)	8 to 12	60 sec	Very hard	2_1	6	Zig-zag drill F (5 cones, 2 m)	20 sec	10 sec	Very hard
	7	Horizontal pull (45 degrees)	8 to 12	60 sec	Very hard	2_1	7	Zig-zag drill B (5 cones, 2 m)	20 sec	10 sec	Very hard
	8	Low-back extensions	8 to 12	60 sec	Very hard	2_1	8	T-drill (10 m–5 m–5 m)	20 sec	10 sec	Very hard
	3 SETS PER STATION						4 rounds with 2 minutes rest between rounds				
WEEK	STRENGTH (HYPERTHROPHY) WORK AT STATIONS						CARDIO-AGILITY				
6		Main part	Tabata Circuit-STRENGTH					Main part	CARDIO-AGILITY TABATA		
	#	Exercises	REPS	REST	RPE	Tempo	#	Exercises	work	REST	RPE
	1	Prisoner squat	8 to 12	60 sec	Very hard	2_1	1	A-skip	20 sec	10 sec	Very hard
	2	Push-up	8 to 12	60 sec	Very hard	2_1	2	Lateral shuffle (5 m–5 m)	20 sec	10 sec	Very hard
	3	Sit-ups	8 to 12	60 sec	Very hard	2_1	3	Backpedal (10 m)	20 sec	10 sec	Very hard
	4	Vertical pull (assisted)	8 to 12	60 sec	Very hard	2_1	4	Carioca (5 m–5 m)	20 sec	10 sec	Very hard
	5	Front lunge (alternate)	8 to 12	60 sec	Very hard	2_1	5	8-turn (10 m left–right)	20 sec	10 sec	Very hard
	6	Shoulder press (floor)	8 to 12	60 sec	Very hard	2_1	6	Zig-zag drill F (5 cones, 2 m)	20 sec	10 sec	Very hard
	7	Horizontal pull (45 degrees)	8 to 12	60 sec	Very hard	2_1	7	Zig-zag drill B (5 cones, 2 m)	20 sec	10 sec	Very hard
	8	Low-back extensions	8 to 12	60 sec	Very hard	2_1	8	T-drill (10 m–5 m–5 m)	20 sec	10 sec	Very hard
	3 SETS PER STATION						4 rounds with 90 seconds rest between rounds				

	WEDNESDAY						THURSDAY				
WEEK	STRENGTH AND LOW IMPACT PLYOMETRICS (COMPLEX)						CARDIO-PACE RUN (INTERVAL)				
5		Main part	Tabata Circuit-STRENGTH					Main part	CARDIO-AGILITY TABATA		
	#	Exercises	REPS	REST	RPE	Tempo	#	Exercises or Activity	PACE	REST	RPE
	1	Air squat + squat jump	5 + 5	30 sec	Very hard	1_1	1	Interval run 8 x 400 METERS	1:30-1:50 (min:sec) per Interval	2 minutes between intervals	Very hard
					Very hard	1_1					
	2	Push-up + plyo push-up	5 + 5	30 sec	Very hard	1_1					
					Very hard	1_1					
	3	Side lunge + lateral bounds	6 + 6	30 sec	Very hard	1_1					
					Very hard	1_1					
	4	Sit-ups + V-ups	5 + 5	30 sec	Very hard	1_1					
					Very hard	1_1					
	5 rounds with 2 minutes rest between rounds										
WEEK	STRENGTH AND LOW IMPACT PLYOMETRICS (COMPLEX)							CARDIO-STEADY STATE			
6		Main part	Tabata Circuit-STRENGTH				#	Exercises	TIME		Tempo
	#	Exercises	REPS	REST	RPE	Tempo	1	STEADY-STATE RUNAT 80-90% OF MAXIMAL HEART RATE IN OUTDOOR CONDITIONS	30 Minutes		Very hard
	1	Air squat + squat jump	6 + 6	30 sec	Very hard	1_1					
					Very hard	1_1					
	2	Push-up + plyo push-up	6 + 6	30 sec	Very hard	1_1					
					Very hard	1_1					
	3	Side lunge + lateral bounds	8 + 8	30 sec	Very hard	1_1					
					Very hard	1_1					
	4	Sit-ups + V-ups	6 + 6	30 sec	Very hard	1_1					
					Very hard	1_1					
	6 rounds with 2 minutes rest between rounds										
